# Primary malignant tumors of the trachea: a retrospective analysis of the clinical data of 79 patients treated in a single center

**DOI:** 10.3389/fonc.2025.1568589

**Published:** 2025-04-09

**Authors:** Qiuyan Chen, Keying Xue, Yigen Wu, Bingqing Luo, Yuyi Lin

**Affiliations:** ^1^ Department of Radiation Oncology, The Second Affiliated Hospital of Xiamen Medical College, Xiamen, Fujian, China; ^2^ Department of Respiratory Centre, The Second Affiliated Hospital of Xiamen Medical College, Xiamen, Fujian, China; ^3^ Department of Thoracic Surgery, The Second Affiliated Hospital of Xiamen Medical College, Xiamen, Fujian, China

**Keywords:** tumors, trachea, ACC, SCC, os

## Abstract

**Background:**

Primary malignant tumors of the trachea are rare. There are few data on such tumors, the understanding of the disease is limited, and the best treatment plan has not yet been determined.

**Methods:**

Clinical data obtained from the medical records of 79 patients with primary malignant tumors of the trachea treated in our hospital between August 2008 and August 2023 were retrospectively analyzed. The clinical data included demographic characteristics (age, sex), carcinogen exposure (smoking or drinking), symptoms, histology, primary tumor location (cervical trachea, intrathoracic trachea or bronchus), primary tumor range, lymph node status, and treatment. SPSS 26.0 software was used for statistical analysis. The Kaplan– Meier method was used to calculate the survival rate, and the log-rank test was used to compare the survival differences between groups. Multivariate analysis was performed using the Cox regression model.

**Results:**

Patients with primary tracheal ACC were significantly younger than those with SCC were (45.5 years old vs. 66.0 years old, P = 0.000007). SCC is more common in smoking and male patients, whereas ACC and other pathological types are more common in nonsmoking and female patients. ACC patients were less likely to have lymph node metastasis than SCC patients were (12.5% vs. 36%, P = 0.047). The 3-year, 5-year and 10year overall survival rates were 69.9%,62.3% and 34.2%, respectively, and the median OS was 96 months. The 3-year overall survival rates of patients with ACC, SCC, and other pathological types were 86.3%, 47.1%, and 71.4%, respectively. The 5-year overall survival rates were 77.0%, 26.5% and 62.5%, respectively. The 10-year overall survival rates were 39.5%, 13.3% and 62.5%, respectively. The overall survival of SCC patients was the shortest among all pathological types, and the difference was statistically significant. The COX regression analysis further demonstrated that a higher N stage is significantly associated with an elevated risk of distant metastasis.

**Conclusion:**

Primary malignant tumors of the trachea are rare, and the best treatment has not yet been determined. Although most patients in this center are treated via a variety of methods, whether this varied approach to treatment is the reason for the higher overall survival cannot be ascertained. Moreover, most patients in our center received a variety of treatments, so a survival analysis of specific treatment modalities was not possible. Thus, more studies involving more patients are needed to ascertain the optimal treatment plan for malignant tracheal tumors.

## 1 Introduction

As primary malignant tumors of the trachea are rare, there are few data on such tumors. Only 0.2 cases are diagnosed per 100,000 people per year (1–4). Malignancies of the larynx and bronchi are observed roughly 40 and 400 times more often than tracheal cancer, respectively (5). The lower occurrence of squamous cell carcinoma in the trachea compared to laryngeal and bronchial cancers is likely due to the trachea’s laminar airflow, which may reduce the accumulation of carcinogens on the tracheal mucosa (5). An increased incidence of tracheal cancer was linked to elevated levels of smoking, alcohol consumption, diabetes, lipid metabolism disorders, and higher HDI (6). The main histological types are adenoid cystic carcinoma (40%) and squamous cell carcinoma (36%) (7). The symptoms associated with tracheal tumors are usually similar to those of head and neck tumors, such as hemoptysis, sore throat and airway obstruction (8). The most common symptom is dyspnea, followed by hemoptysis and cough (2, 9, 10). Hemoptysis is more common in patients with squamous cell carcinoma, and dyspnea is more common in patients with adenoid cystic carcinoma (11). Chronic cough is usually considered related to asthma or, in smokers, related to chronic lung disease. The unclear natural history and unspecific symptoms of tracheal tumors, as well as their slow growing nature, lead to tracheal malignant tumors not being diagnosed for several months to several years (2). The average duration of symptoms before diagnosis is generally 4 months for squamous cell carcinoma and 12 months for adenoid cystic carcinoma (10).

Among tracheal cancer patients, 25.7%-40% are diagnosed with adenoid cystic carcinoma, and 25.7%-45.2% have squamous cell carcinoma (2, 7, 10, 12). The remaining histological types are diverse and include squamous papillary carcinoma, carcinoid tumors, mucoepidermoid tumors, and various cancers and sarcomas (12, 13). Five hundred seventy-eight cases were registered in the Surveillance Epidemiology End Results (SEER) database from 1973 to 2004, making primary malignant tumors of the trachea the most widely reported malignant tumors. Squamous cell carcinoma was the main histological type (45%), followed by adenoid cystic carcinoma (16.3%), undifferentiated carcinoma (12.8%), small cell carcinoma (9.7%), adenocarcinoma (5.9%), large cell carcinoma (3.8%) and sarcoma (3.8%) (14, 15).

Primary tracheal tumors are rare, and the understanding of the disease is limited. The best treatment plan has not yet been determined (16–21). There are three main methods for treating tracheal tumors: tracheal resection, endoscopic resection and radiotherapy. When possible, surgical resection with or without radiotherapy is considered the best method for long-term survival (2, 10–12, 22). Surgical treatment is the first choice for early treatment. Extensive resection can control the disease, and the 5-year survival rate is 20%-40%for patients with squamous cell carcinoma and 60%-100%for patients with adenoid cystic carcinoma (4, 7). In cases where radical surgery is not possible, different endoscopic treatments or radiotherapy can be used (23). External irradiation has been widely used in the palliative treatment of tracheal cancer. Numerous studies have confirmed the value of radiotherapy in the control of primary tracheal tumors. Seventy-five percent of patients experience remission, and the 5-year survival rate can reach 10% (18, 23, 24). Intracavitary irradiation has also been shown to be safe and effective in the palliative treatment of inoperable tracheal tumors (25). Moreover, intracavitary brachytherapy, which involves the insertion of radioactive agents via an applicator, can control tumors without damaging the surrounding normal tissues (21, 23, 26).

## 2 Materials and methods

A retrospective analysis of the clinical data of 79 patients with primary malignant tumors of the trachea treated in our hospital from August 2008 to August 2023 was performed. All patients were diagnosed with tracheal malignant tumors by histopathology. Among the 79 patients, 52 were treated for new cases (newly diagnosed, previously untreated), 12 were treated for recurrent cases, 5 were treated for obvious residual or persistent tumors, 7 were treated for scar stenosis, and 3 underwent stent removal, stent replacement and tracheoesophageal fistula creation.

Clinical data were collected from the medical records database center. Clinical data, including demographic characteristics (age, sex), carcinogen exposure (smoking and drinking), symptoms, histology, primary tumor location (cervical trachea, intrathoracic trachea and bronchus), primary tumor range, lymph node status, treatment and survival results, were analyzed.

For primary malignant tumors of the trachea, neither UICC staging nor American Joint Committee on Cancer (AJCC) staging data were available (2, 27). Therefore, preoperative imaging data were used for retrospective staging. The location and extent of the tumor were estimated on computed tomography (CT) and bronchoscopy images. The primary tumor stage (T) was described as E1(the tumor is confined to the trachea or has spread beyond the trachea but has not reached the adjacent organs) or E2(the tumor has spread to the adjacent organs or other structures). The lymph node status (N)was described as N0 (no lymph node metastasis) or N1(lymph node metastasis). Distant metastasis (M) was either M0 (no distant metastasis) or M1 (distant metastasis) (28–31).

SPSS 26.0 software was used for statistical analysis. The Kaplan–Meier method was used to calculate the survival rate, and the log-rank test was used to compare the differences between groups, with a P value < 0.05 indicating that the difference was statistically significant.

## 3 Results

A total of 79 patients with primary malignant tumors of the trachea were admitted to our hospital between August 2008 and August 2023. There were 39 males and 40 females. Among the 79 patients with primary malignant tumors of the trachea, 32 (40.5%) were ACC patients, and 25 (31.6%) were SCC patients. The remaining histological types were defined as ‘Other’ and were not specified. Among these other pathological types, well-differentiated leiomyosarcoma was found in 1 patient (1.3%), sarcomatoid carcinoma in 2 patients (2.5%), small cell carcinoma in 1 patient (1.3%), large cell neuroendocrine carcinoma in 2 patients (2.5%), acinar cell carcinoma in 4 patients (5.1%), lymphoepithelioma-like carcinoma in 2 patients (2.5%), adenosquamous carcinoma in 3 patients (3.8%), cystadenocarcinoma in 1 patient (1.3%), carcinoid carcinoma in 1 patient (1.3%), squamous carcinogenesis of the mucosal epithelium in 1 patient (1.3%), mucoepidermoid carcinoma in 2 patients (2.5%), and adenocarcinoma in 2 patients (2.5%). There are few records of the specific pathological grading.

The clinical and demographic data of these 79 patients with primary tracheal cancer are shown in **Table 1**. The age at onset ranged from 6 to 84 years, with a median age of 51 years. The median age at onset was 56 years for men and 44 years for women. The median age of the ACC patients was 45.5 years, and the median age of the SCC patients was 66 years.

**Table 1:** Demographic and clinical characteristics of 79 patients with tracheal cancers by tumor histology.

Variable	SCC (n=25)	ACC		Other	
		n=32	P value*	n=22	P value*
Age,y, mean ± SD (median)	64.1 ± 12.3 (66.0)	46.7 ± 13.8 (45.5)	0.000007	46.3 ± 20.9 (50.0)	0.001
Sex,n (%)					
Male	18 (72.0)	13 (40.6)	0.018	8 (36.4)	0.014
Female	7 (28.0)	19 (59.4)		14 (63.6)	
Smoking,n (%)					
Never	9 (36.0)	22 (68.8)	0.008	16 (72.7)	0.004
Sometimes	1 (4.0)	2 (6.3)		2 (9.1)	
Always	15 (60.0)	8 (25.0)		4 (18.2)	
Drinking,n (%)					
Never	18 (72.0)	27 (84.4)	0.127	17 (77.3)	0.311
Sometimes	4 (16.0)	5 (15.6)		5 (22.7)	
Always	3 (12.0)	0 (0)		0 (0)	
Location,n (%)					
Cervical	4 (16.0)	7 (21.9)	0.585	5 (22.7)	0.569
Thoracic	21 (84.0)	25 (78.1)		17 (77.3)	

*t-test comparing the mean age of the patients or chi-square analysis comparing the proportion of patients with ACC or other histological types in each category with that of patients with SCC.

More than half of the SCC patients were male, and most ACC patients were female. Most SCC patients were smokers, and most ACC patients were never smokers. The most common disease location was the thoracic trachea.

These patients’ disease staging results are shown in **Table 2**. Most cases were limited to the trachea and did not invade the adjacent trachea, which was similar across different pathological types. Although most patients had no lymph node metastasis, ACC patients were less likely to have lymph node metastasis than SCC patients, and the difference was statistically significant (P = 0.047). Most patients had no distant metastasis at the time of treatment.

**Table 2:** Staging and treatment of the 79 patients with tracheal cancers.

Variable	ACC (n=32)		SCC (n=25)			Other (n=22)		
	n	%	n	%	P value*	n	%	P value*
T stage					0.436			0.326
E1	27	84.4	19	76.0		16	72.7	
E2	5	15.6	6	24.0		6	27.3	
N stage					0.047			0.905
N0	28	87.5	16	64.0		19	86.4	
N1	4	12.5	9	36.0		3	13.6	
M stage					0.802			0.793
M0	30	93.8	23	92.0		21	95.5	
M1	2	6.3	2	8.0		1	4.5	

*t-test comparing the mean age of the patients or chi-square analysis comparing the proportion of patients with ACC or other histological types in each category with that of patients with SCC.

Among these patients, the most common symptoms were cough (87.3%), asthma (84.8%), hemoptysis (26.6%), chest tightness (27.8%), dyspnea (5.1%), dysphagia (3.8%), hoarseness (1.3%), substernal pain (2.5%), and coughing (2.5%).

Most of the 79 patients underwent a varied treatment approach, whereas only a few patients were treated via a single treatment method. The treatment methods included the following: tracheoscopic resection (44 cases; 55.7%);ablation, including argon-helium knife, helium knife, argon knife, Haibo knife, lesion cryotherapy, microwave ablation, and electric knife cauterization (41 cases;51.9%);immunotherapy (8 cases; 10.1%);radioactive seed implantation (22 cases; 27.8%);external irradiation radiotherapy (27 cases; 34.2%);chemotherapy (32 cases; 40.5%);surgical resection (11 cases; 13.9%);photodynamic therapy (4 cases; 5.1%);targeted therapy (3 cases; 3.8%); and bronchial artery embolization (1 case; 1.3%). Among these patients, 9 (11.4%) underwent endoscopic resection, 3 (3.8%) underwent ablation, 1 (1.3%) underwent radioactive seed implantation, 1 (1.3%) underwent chemotherapy, and 2 (2.5%) underwent surgical resection. The remaining patients (63 patients, 79.7%) received multiple treatments.

Among the patients who underwent ablation, some were treated with one ablation treatment, and some received multiple ablation treatments. Five patients (6.3%) underwent argon-helium knife ablation, 2 patients (2.5%) were treated with helium knife ablation, 11 patients (13.9%) were treated with argon knife ablation, 1 patient (1.3%) was treated with Haibo knife ablation, 29 patients (36.7%) underwent lesion freezing, 2 patients (2.5%) were treated with microwave ablation, and 17 patients (24.6%) were treated with electric knife ablation.

Eight patients received immunotherapy, and the drugs used were sindilizumab (5 patients), carrelizumab (2 patients), and tirelizumab (1 patient). Of the 8 patients who received immunotherapy, 6 were treated after the initial diagnosis, 1 after metastasis, and 1 after disease recurrence.

Iodine 125 was used for radioactive particle implantation in 22 patients, 8 of whom were directly implanted with the radioactive particles, and the remaining 14 were implanted with radioactive stents. The implantation sites were the trachea (19 patients), lung (1 patient with lung metastasis), liver (1 patient with liver metastasis) and carina (1 patient with carina metastasis). The activity of radioactive particles is 0.5–0.6 mCi.

Among the 27 patients who received external beam radiotherapy, 22 patients received only radical radiotherapy (5 patients received radical radiotherapy due to recurrence of the lesion, and the rest received radical radiotherapy after diagnosis), and 4 patients received concurrent radical radiotherapy (2 patients received 2 concurrent cycles of single-agent cisplatin chemotherapy, 1 patient received 2 concurrent cycles of endostar targeted chemotherapy, and 1 patient received 2 concurrent cycles of docetaxel + vinorelbine chemotherapy). One patient received postoperative adjuvant radiotherapy. The mean dose of radical radiotherapy was 64.4 Gy (range 60–70 Gy). The dose of postoperative adjuvant radiotherapy was unknown.

Among the 32 patients who received chemotherapy, 1 patient was treated with pericardial perfusion chemotherapy due to pericardial metastasis (1 cycle of lobaplatin), 1 patient was treated with chemotherapy due to disease progression (2 cycles of gemcitabine), and the remaining patients were treated with chemotherapy after the diagnosis of the disease. The average number of cycles of chemotherapy was 3.2 (range, 1-8 cycles). The chemotherapeutic drugs used were gemcitabine, cisplatin, lobaplatin, nedaplatin, paclitaxel, vinorelbine, carboplatin, etoposide, docetaxel, pemetrexed, and tegafur. The most commonly used chemotherapeutic drugs are gemcitabine, cisplatin, docetaxel, and paclitaxel, and at least 5 patients were treated with the above chemotherapeutic drugs. At least 10 patients were treated with gemcitabine and cisplatin.

Among the 3 patients who received targeted therapy, 1 patient was treated with anlotinib, and 1 patient was treated with gefitinib first, followed by apatinib combined with gefitinib. One patient was treated with oral apatinib (targeted therapy was suspended due to an oral ulcer).

The follow-up period ended on October 23, 2023. Three patients were lost to follow-up, for a follow-up rate of 96.2%. The median follow-up time was 39.5 months (1–223 months). Tumor-free survival was observed in 28 patients, and 28 cases of recurrence were observed in the whole group. Among them, 18 patients experienced tracheal cancer recurrence, 1 patient experienced mediastinal lymph node cancer recurrence, 2 patients experienced both tracheal and mediastinal lymph node cancer recurrence, and the remaining 7 patients had unknown recurrence sites. There were 20 patients with metastasis in the whole group, including 4 patients with liver metastasis, 11 patients with lung metastasis, 3 patients with bone metastasis, 1 patient with pleural metastasis, 1 patient with retroperitoneal metastasis, 1 patient with pericardial metastasis, 1 patient with adrenal metastasis and 1 patient with supraclavicular lymph node metastasis. Among the 38 patients who died, 34 (89.4%) died from tracheal tumors, and 4 (10.5%) died from other causes.

The 3-year, 5-year and 10-year overall survival rates were 69.9%,62.3% and 34.2%, respectively, and the median OS was 96 months. The 3-year overall survival rates of ACC patients, SCC patients, and patients with other pathological types were 86.3%, 47.1%, and 71.4%, respectively. The 5-year overall survival rates were 77.0%, 26.5% and 62.5%, respectively. The 10-year overall survival rates were 39.5%, 13.3% and 62.5%, respectively. The median OS times of ACC patients and SCC patients were 103 months and 36 months, respectively. The median OS of patients with other pathological types has not yet been calculated.

The 3-year, 5-year and 10-year progression-free survival rates were 58.1%, 35.8% and17.7%, respectively, and the median PFS was 45 months. The 3-year progression-free survival rates of ACC patients, SCC patients, and patients with other pathological types were 71.9%, 47.5%, and 50.8%, respectively. The 5-year progression-free survival rates were 43.1%, 23.8%and 40.6%, respectively. The 10-year progression-free survival rates were 18.5%, 11.9% and has not yet been calculated, respectively. The median PFS of the three groups were 60 months, 36 months and 52 months, respectively.

The 3-year, 5-year and 10-year recurrence-free survival rates were 76.2%, 54.1% and 30.0%, respectively, and the median RFS was 72 months. The 3-year recurrence-free survival rates of ACC patients, SCC patients, and patients with other pathological types were 77.6%, 81.3%, and 73.3%, respectively. The 5-year recurrence-free survival rates were 51.4%, 55.7%, and 61.1%, respectively. The 10-year recurrence-free survival rates were 22.0%, 55.7%, and has not yet been calculated, respectively. The median RFS of ACC patients was 72 months, and the median RFS of SCC patients and patients with other pathological types has not yet been calculated.

The 3-year, 5-year and 10-year distant metastasis-free survival rates were 84.4%, 74.3% and 49.6%, respectively, and the median DMFS was 107 months. The 3-year distant metastasis-free survival rates of ACC patients, SCC patients, and patients with other pathological types were 86.2%, 83.1%, and 83.0%, respectively. The 5-year distant metastasis-free survival rates were 68.6%, 62.3%, and 83.0%, respectively. The 10-year distant metastasis-free survival rates were 38.6%, 62.3%, and 83.0%, respectively. The median DMFS of ACC patients was 100 months, and the median DMFS of SCC patients and patients with other pathological types has not yet been calculated.

The results of the survival analysis for the whole group of patients are shown in **Table 3**.

**Table 3:** Results of the survival analysis for the whole group of patients.

Variable	PFS		OS		RFS		DMFS	
	t (median)*	P value&	t (median )	P value	t (median )	P value	t (median )	P value
Age								
<51	48	0.646	96	0.977	130	0.800	96	0.468
≥51	44		99		72			
Sex								
Male	39	0.164	63	0.320	49	0.251	107	0.705
Female	60		103		130		132	
Smoking								
Never	48	0.345	99	0.275	72	0.797	132	0.519
Sometimes	52		67		52			
Always	36		36		107		107	
Drinking								
Never	49		99	0.000	76		132	0.915
Sometimes	44	0.002	63	164	52	0.950	107	
Always	5		5					
Location								
Cervical	37	0.997	120	0.192	76	0.993		0.460
Thoracic	45		67		72		107	
T stage								
E1	44	0.867	99	0.830	72	0.673	107	0.705
E2	52		84		76			
N stage								
N0	49	0.066	103	0.102	72	0.835		0.003
N1	26		67		107		61	
M stage								
M0	48	0.021	96	0.075	72	0.328	132	0.019
M1	5		10		107		107	
Histology								
SCC	36	0.181	36	0.003		0.911		0.502
ACC	60		103		72		100	
Other	52							

*Fields with missing data (the median)have not yet been calculated.

&Differences between groups were compared using the log-rank test.

**Figure 1:**
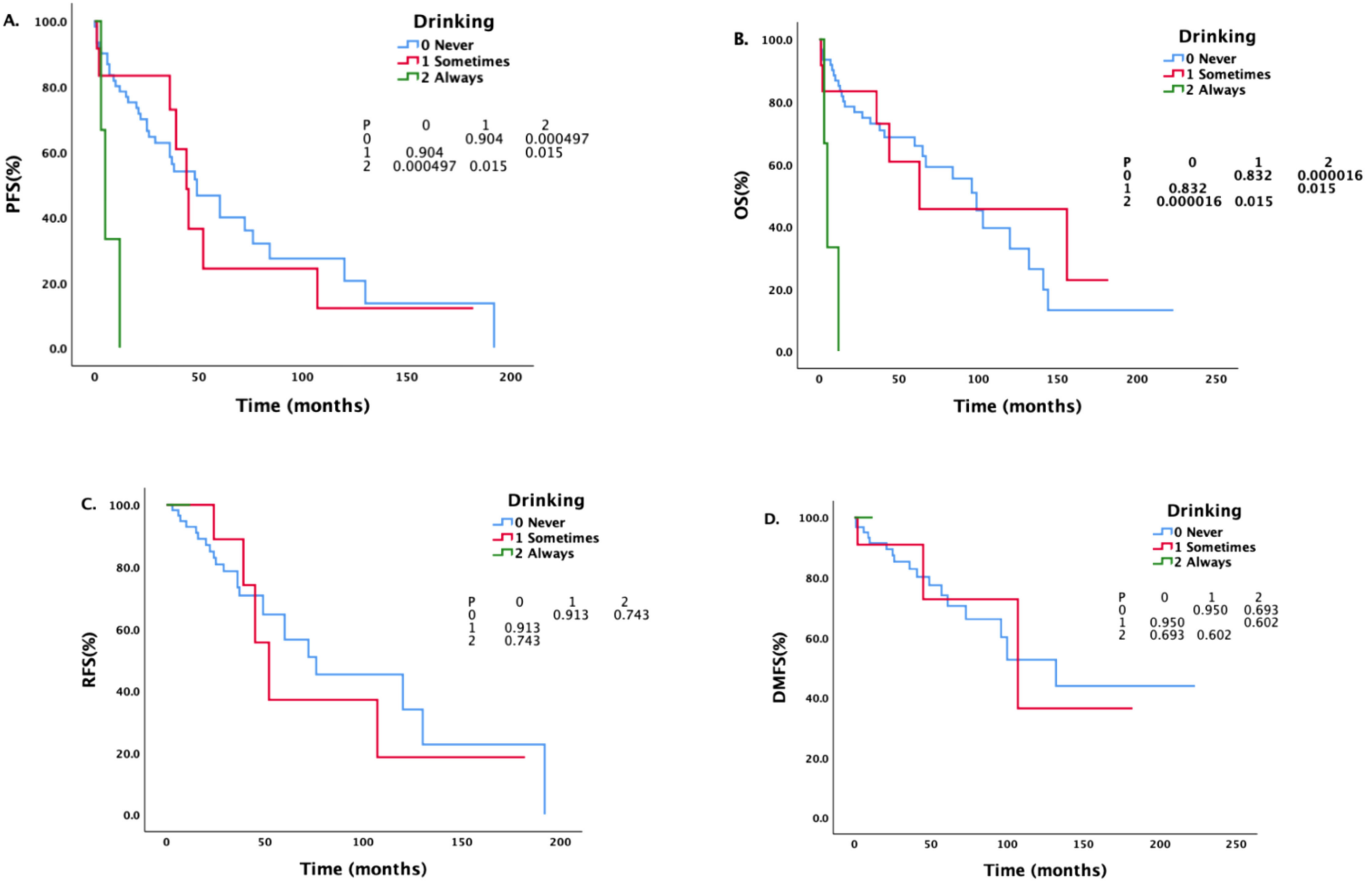
Survival analysis and comparison of patients with different drinking histories. The impact of different drinking statuses on survival prognosis.

Patients with different drinking histories had statistically significant differences in PFS and OS (see **Figure 1**). The median PFS of never drinkers, occasional drinkers, and regular drinkers were 49 months, 44 months, and 5 months, respectively. There was a statistically significant difference in PFS between never drinkers and regular drinkers (P = 0.000497). The difference in PFS between occasional and regular drinkers was statistically significant (P = 0.015). There was no significant difference in PFS between never drinkers and occasional drinkers (P = 0.904). The median OS of never drinkers, occasional drinkers, and regular drinkers were 99 months, 63 months, and 5 months, respectively. There was a statistically significant difference in OS between never drinkers and regular drinkers (P = 0.000016), and there was a statistically significant difference in OS between occasional drinkers and regular drinkers (P = 0.015). There was no significant difference in OS between never drinkers and occasional drinkers (P = 0.832).

**Figure 2:**
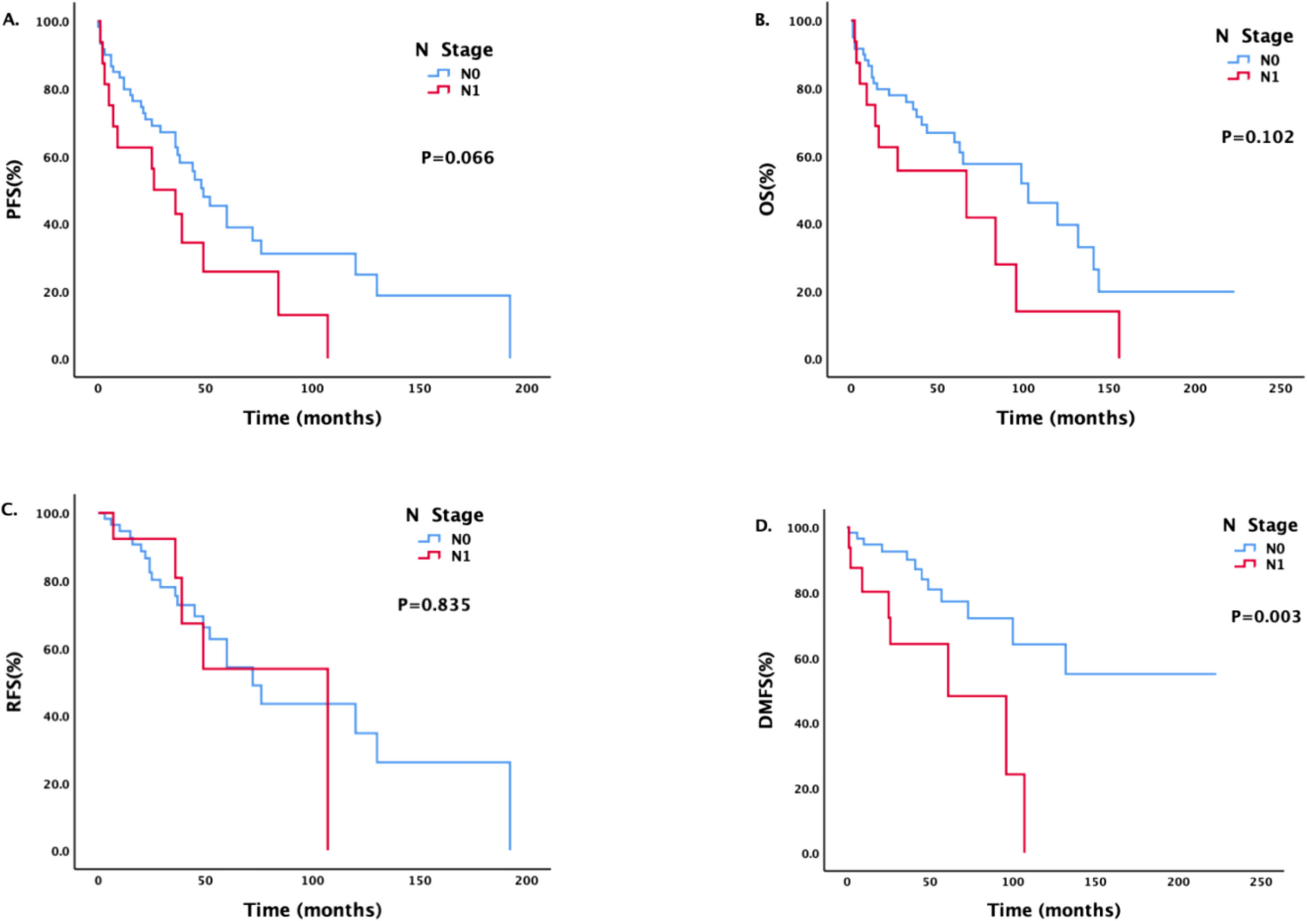
Survival analysis and comparison of patients with different N stages. Impact of lymph node status on survival prognosis.

There was a statistically significant difference in DMFS between patients with different N stages (**Figure 2**). The median DMFS of patients with newly diagnosed N0 disease has not yet been calculated, and the median DMFS of patients with N1 disease was 61 months; the difference was statistically significant (P = 0.003).

**Figure 3:**
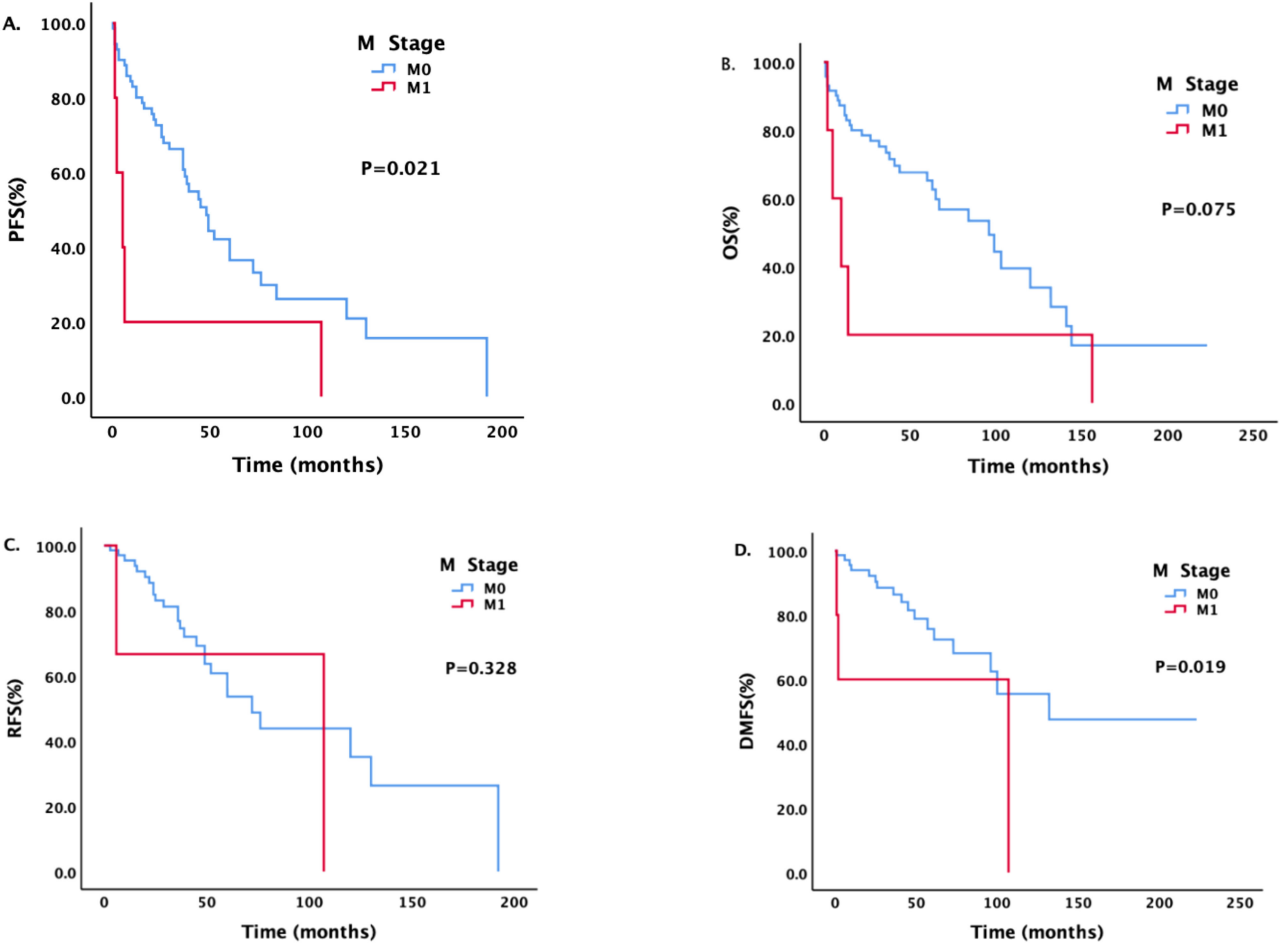
Survival analysis and comparison of patients with different M stages. Impact of distant metastasis on survival prognosis.

Patients with different M stages had statistically significant differences in PFS and DMFS (**Figure 3**). The median PFS of patients without metastasis was 48 months, and the median PFS of patients with metastasis was 5 months; the difference was statistically significant (P = 0.021). The median DMFS of newly diagnosed patients without metastasis was 132 months, and the median DMFS of patients with metastasis was 107 months; the difference was statistically significant (P = 0.019).

**Figure 4:**
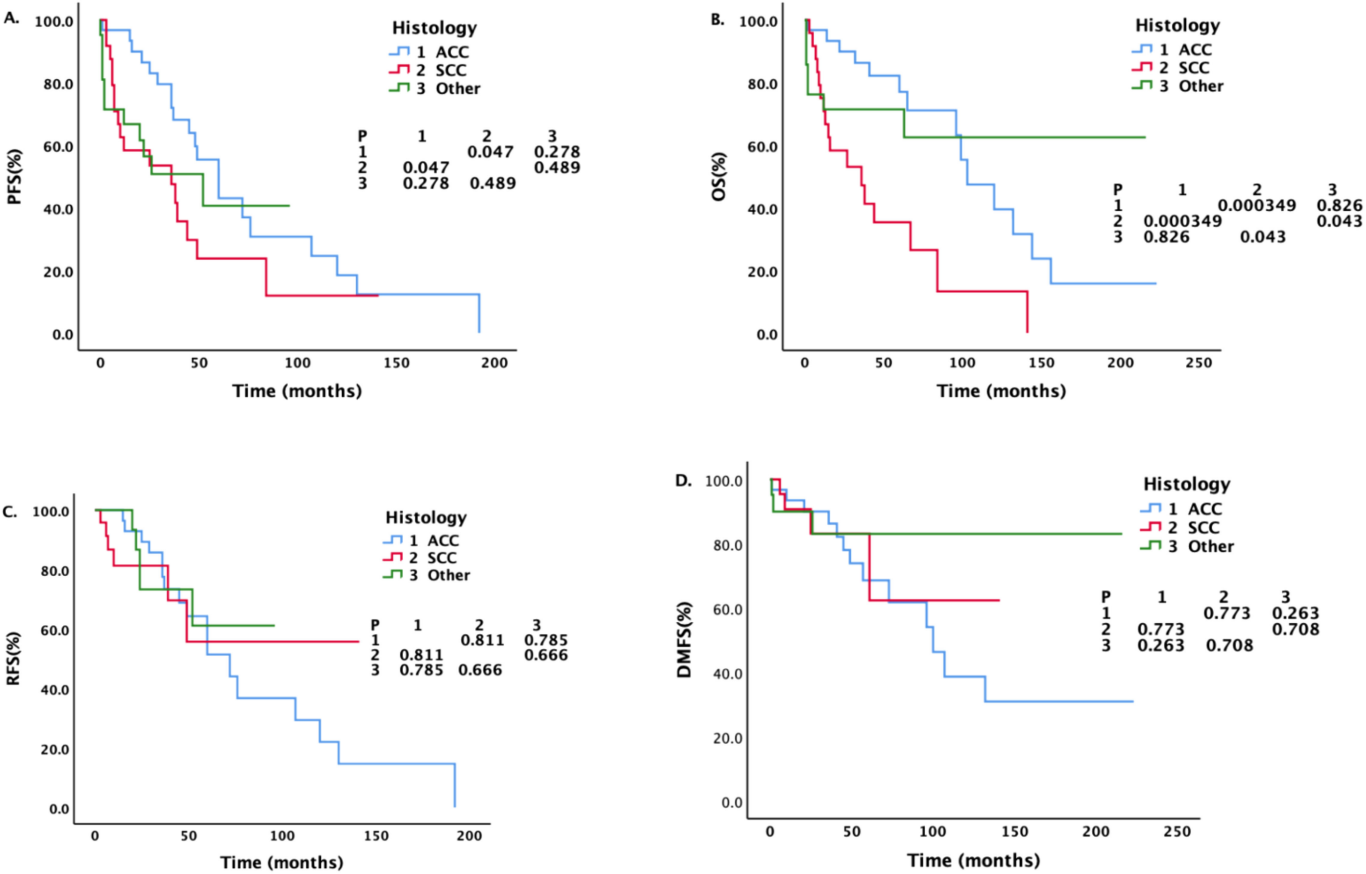
Survival analysis and comparison of patients with different pathological types. Impact of histopathological characteristics on survival prognosis.

**Figure 4** shows that the median PFS of ACC patients was longer than that of SCC patients (60 months vs.36 months, P = 0.047). The median OS of SCC patients was the shortest among all pathological types, whether compared with ACC patients (36 months vs.103 months, P = 0.000349) or patients with other pathological types (P = 0.043). There was no significant difference between patients with ACC and patients with other pathological types (P = 0.826).

**Figure 5:**
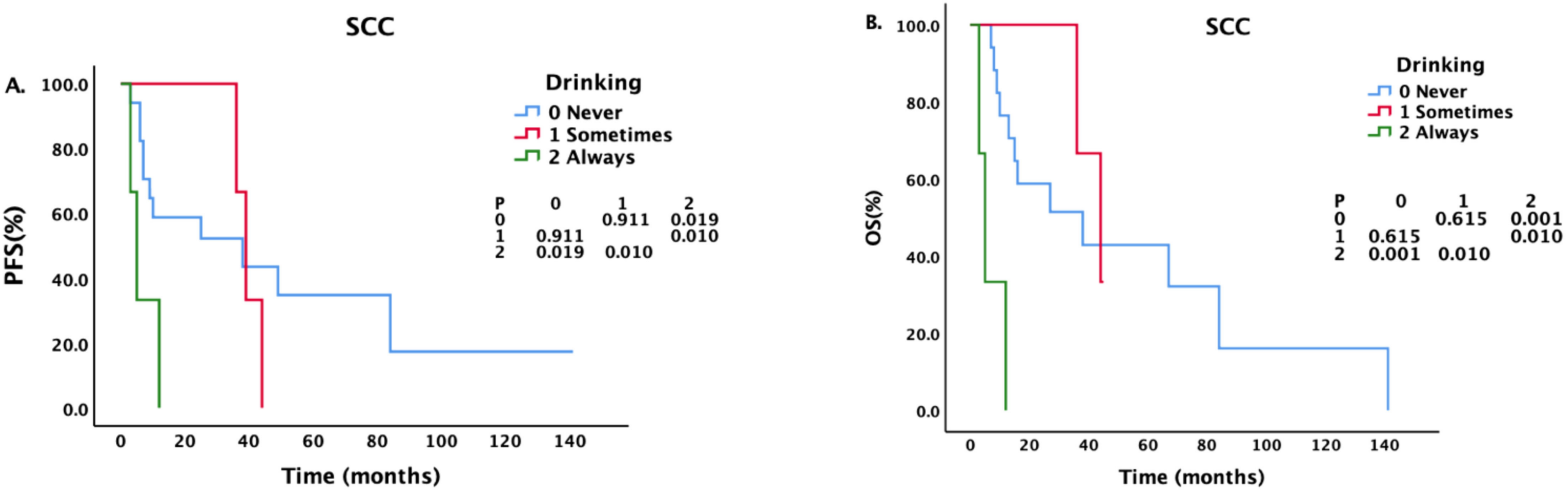
Effects of different drinking statuses on survival in patients with SCC.

**Figure 6:**
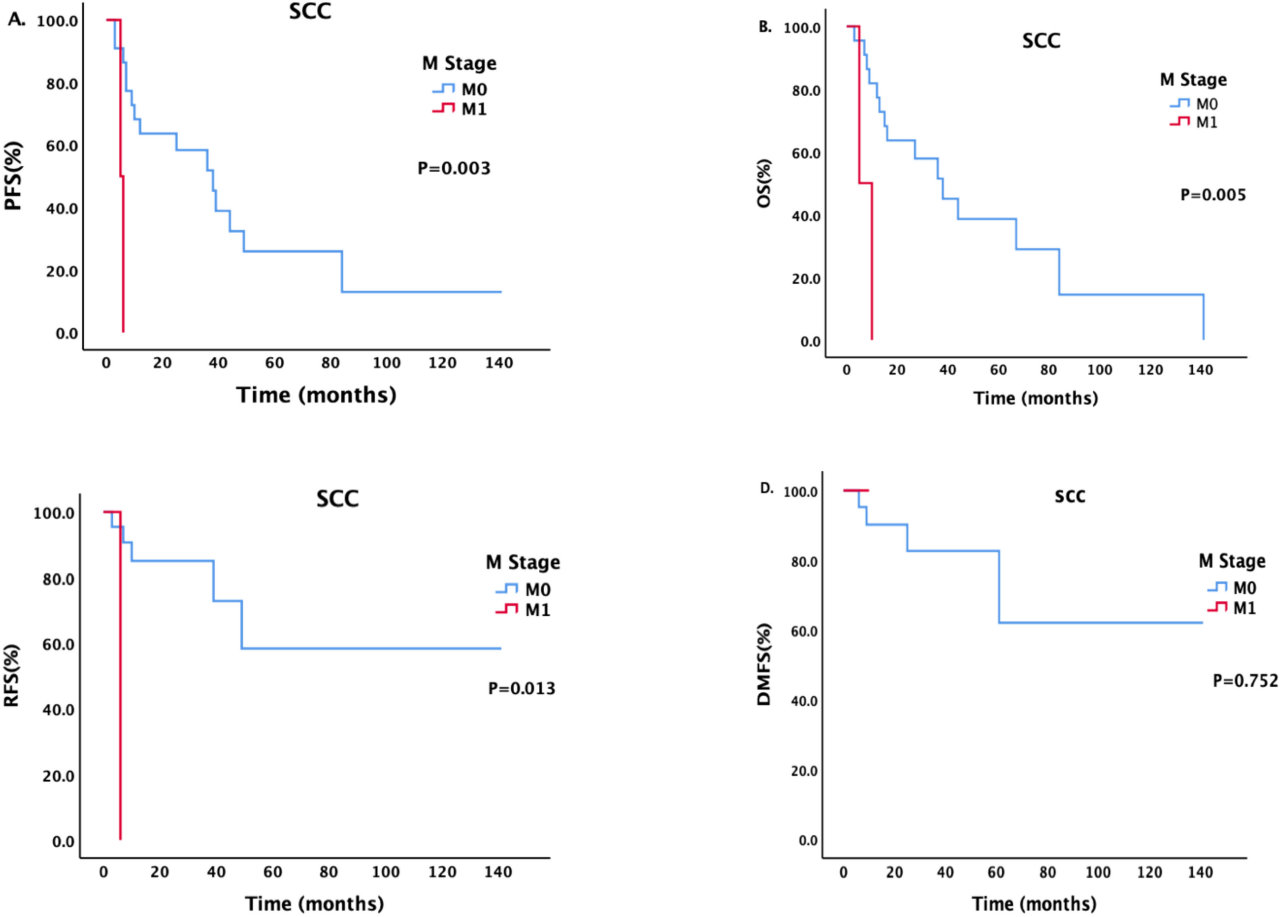
Effects of different M stages on survival in patients with SCC.

**Figure 7:**
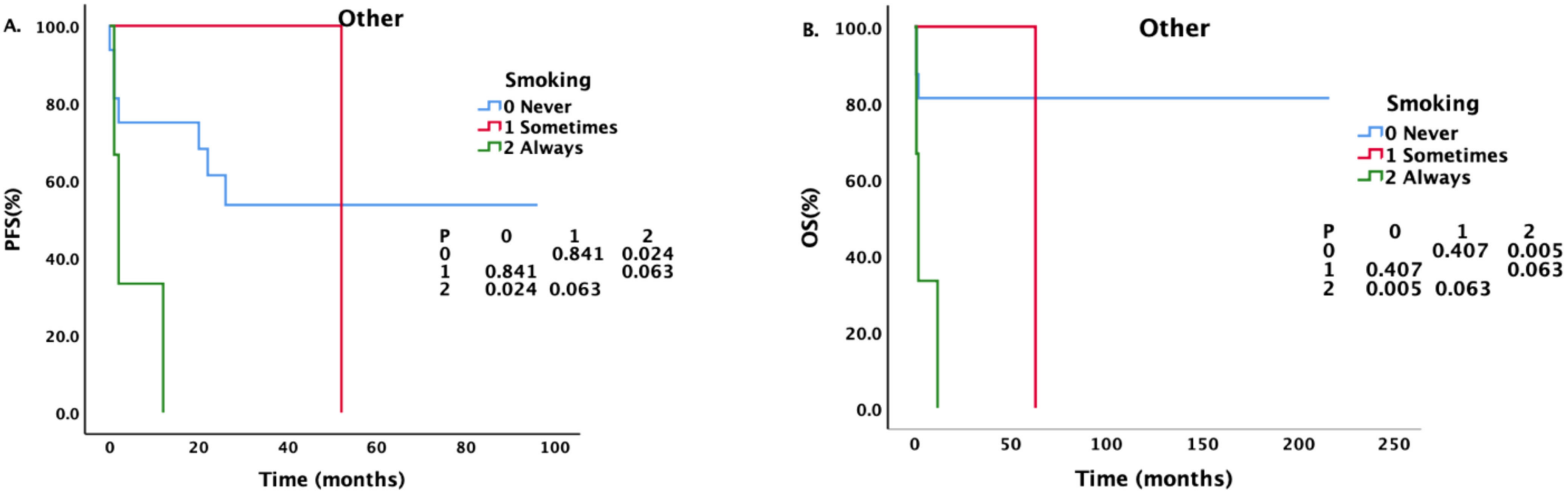
Effects of different smoking statuses on PFS and OS in patients with other pathological types.

**Figure 8:**
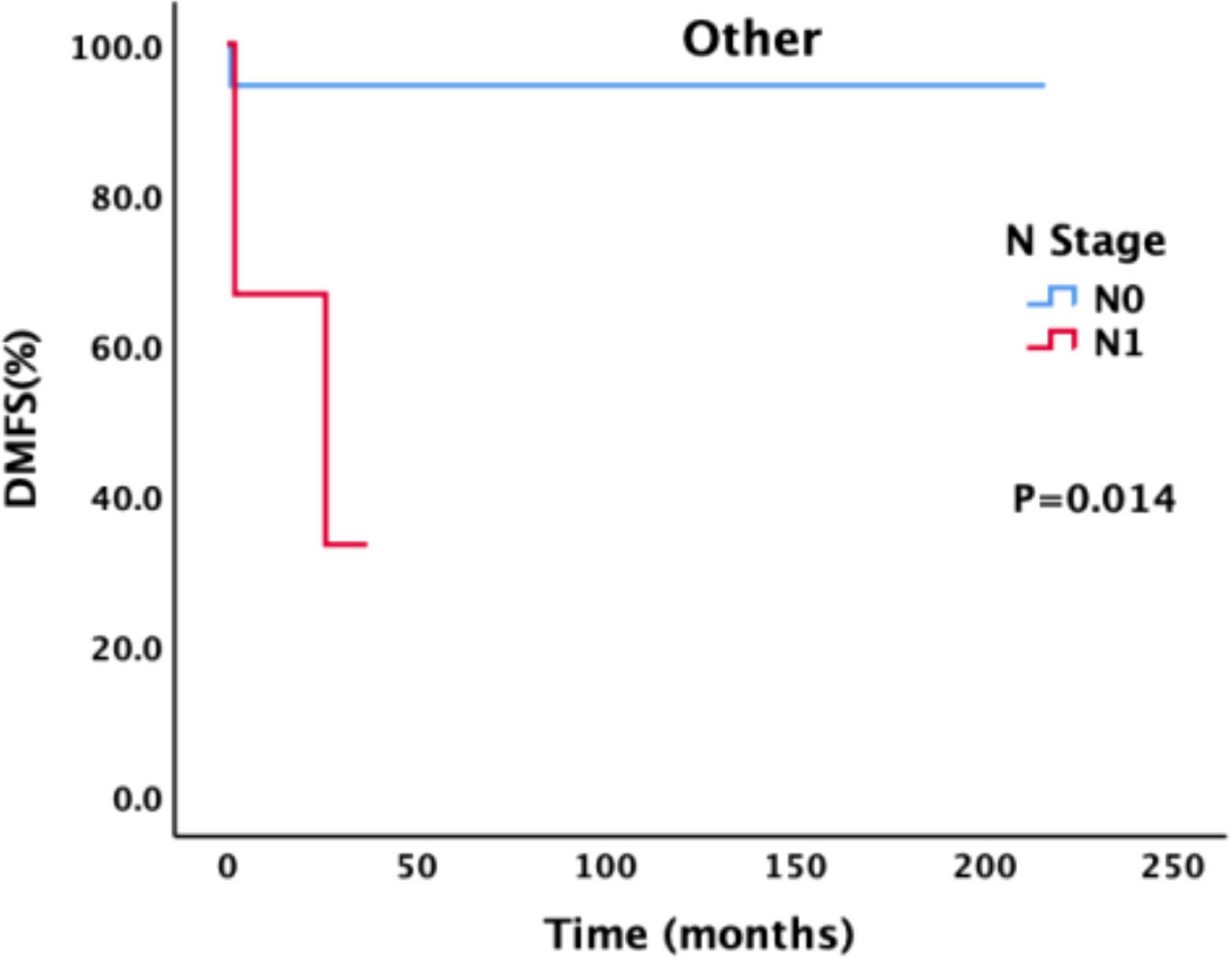
Effect of different N stages on DMFS in patients with other pathological types.

**Figure 9:**
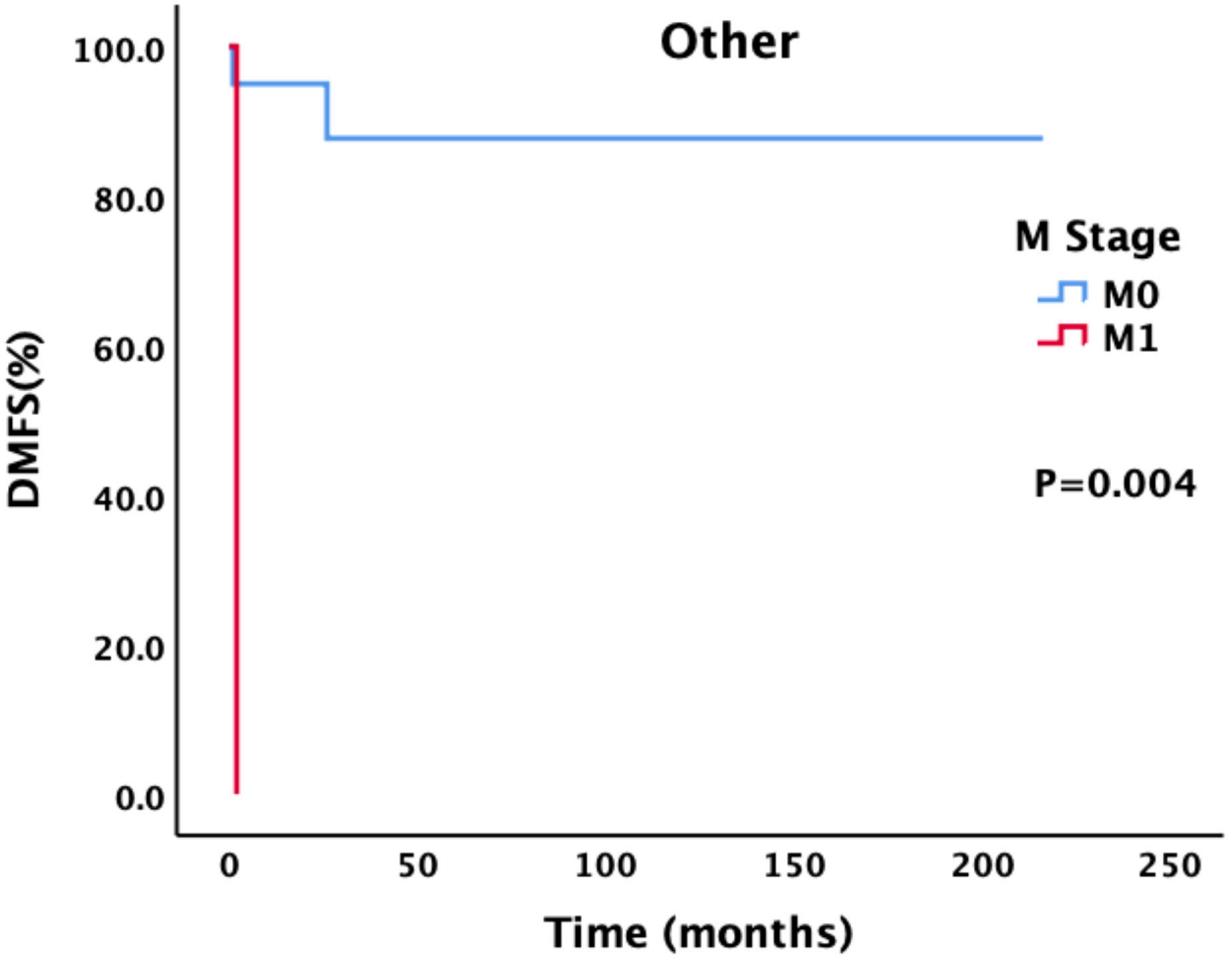
Effect of different M stages on DMFS in patients with other pathological types.

For patients with different pathological types, we also conducted a corresponding analysis (**Tables 4**–**6**). Sex, smoking status, drinking status, T stage, N stage and M stage had no effect on the survival of patients with pathological type ACC (**Table 4**). However, among patients with SCC (**Table 5**), those who drank regularly had significantly shorter PFS and OS than those who did not drink or occasionally drank did (**Figure 5**). The PFS, OS and RFS of patients with metastasis were significantly shorter than those of patients without metastasis (**Figure 6**). Among patients with other pathological types (**Table 6**), those who smoked regularly had significantly shorter PFS and OS than patients who did not smoke did (**Figure 7**). The DMFS of patients with N1 and M1 disease was significantly shorter than that of patients with N0 and M0 disease (**Figures 8**, **9**).

**Table 4:** Survival analysis results for ACC patients.

Variable	PFS		OS		PFS		DMFS	
	t (median)*	P value&	t (median)	P value	t (median)	P value	t (median)	P value
Sex								
Male	49	0.998	144	0.347	76	0.915	73	0.563
Female	60		96		60		100	
Smoking								
Never	60	0.881	99	0.626	60	0.882		0.299
Sometimes	76		144		76			
Always	45		156		107			
Drinking								
Never	60	0.317	99	0.081		0.430		0.817
Sometimes	107		156					
Always	60	0.623	103	0.971	60	0.900	100	
T stage								
E1								0.795
E2	76				76			
N stage								
N0	60	0.576	120	0.646		0.880	100	0.340
N1	36		96				96	
M stage								
M0	60	0.539	103	0.820		0.890		0.244
M1	1		14					

*Fields with missing data (e.g., the median) have not yet been calculated, and some cases were excluded, so the statistics were not calculated.

**Table 5:** Survival analysis results for SCC patients.

Variable	PFS		OS		RFS		DMFS	
	t (median)*	P value&	t (median)	P value	t (median)	P value	t (median)	P value
Sex								
Male	38	0.699	38	0.750	49	0.902	61	0.497
Female	25		27					
Smoking								
Never	25	0.950	27	0.964		0.803		0.487
Sometimes	49		67		49			
Always	36		36					
Drinking								
Never	38		38	0.000				0.619
Sometimes	39	0.019	44	482		0.844		
Always	5		5					
T stage								
E1	36	0.725	36	0.889		0.579		0.945
E2	25		27		39			
N stage								
N0	36	0.388	36	0.604		0.496		0.144
N1	25		27				61	
M stage								
M0	38	0.003	38	0.005		0.013		0.752
M1	5		5					

*Fields with missing data (e.g., the median) have not yet been calculated, and some cases were excluded, so the statistics were not calculated.

**Table 6:** Other survival analysis results for patients with different pathological types.

Variable	PFS		OS		RFS		DMFS	
	t (median)*	P value&	t (median)	P value	t (median)	P value	t (median)	P value
Sex								
Male	22	0.342	63	0.137	52	0.123		0.975
Female								
Smoking								
Never		0.045		0.009		0.341		0.252
Sometimes	52		63		52			
Always	2		2		24			
Drinking								
Never		0.422		0.051		0.282		0.452
Sometimes	2		2					
Always								
T stage								
E1	22	0.549		0.941		0.368		0.221
E2	52		63					
N stage								
N0	52	0.724		0.989		0.339		0.014
N1	26						26	
M stage								
M0	52	0.193		0.113				0.004
M1	2		2					

*Fields with missing data (e.g., the media) have not yet been calculated, and some cases were excluded, so the statistics were not calculated.

&Differences between groups were compared via the log-rank test.

According to the COX regression analysis of the all patients presented in **Table 7**, the N stage is identified as a prognostic factor, with a higher N stage being significantly associated with an increased risk of metastasis.

**Table 7:** COX regression analysis of survival outcomes for all patients.

	PFS		OS		RFS		DMFS	
Variable	HR (95% CI)	P value	HR (95% CI)	P value	HR (95% CI)	P value	HR (95% CI)	P value
Age	0.925 (0.445-1.921)	0.834	0.835 (0.370-1.888)	0.666	0.635 (0.238-1.690)	0.363	0.640 (0.207-1.977)	0.438
Sex	0.687 (0.252-1.872)	0.463	1.057 (0.320-3.489)	0.927	0.379 (0.110-1.306)	0.124	0.814 (0.149-4.444)	0.812
Smoking	0.902 (0.484-1.681)	0.745	1.230 (0.590-2.567)	0.581	0.833 (0.375-1.851)	0.654	1.301 (0.448-3.777)	0.628
Drinking	1.209 (0.513-2.846)	0.664	1.218 (0.484-3.061)	0.676	0.696 (0.179-2.709)	0.602	0.198 (0.026-1.487)	0.115
T stage	0.684 (0.300-1.559)	0.366	0.823 (0.310-2.187)	0.696	0.886 (0.293-2.682)	0.830	0.950 (0.274-3.296)	0.936
N stage	1.631 (0.692-3.845)	0.264	1.501 (0.564-3.994)	0.415	1.000 (0.265-3.778)	1.000	3.338 (1.113-10.008)	0.031
M stage	1.940 (0.529-7.118)	0.318	1.379 (0.348-5.458)	0.647	2.184 (0.256-18.602)	0.475	3.560 (0.463-27.379)	0.222
Histology	1.372 (0.933-2.017)	0.108	1.290 (0.849-1.962)	0.233	0.954 (0.571-1.596)	0.859	0.777 (0.407-1.483)	0.445

## 4 Discussion

During the past 15 years, 79 patients with primary malignant tumors of the trachea were admitted to our hospital. In a study by Hermes C. Grillo (32), 75% of the patients included were diagnosed with either ACC or SCC, and, in our study, 72.1% of the patients were diagnosed with ACC (40.5%) or SCC (31.6%). The patients with primary tracheal ACC (median age, 45.5 years) were significantly younger than those with SCC were (median age 66.0 years), which is consistent with previous reports (2, 7, 28, 33, 34). In addition, in our study, we found that SCC was more common in patients who smoked regularly and were male, whereas ACC was more common in those who were nonsmokers and female, which was consistent with the findings of the study by Benjamin D Webb et al (2, 11, 29, 35). Other pathological types of primary malignant tumors of the trachea are also more common in women and nonsmokers. There was no significant difference in the location of the malignant tracheal tumors according to pathological type. According to the staging of the disease, ACC patients are less likely to have lymph node metastasis than SCC patients are, which is consistent with previous reports (1, 4, 36, 37).

Primary malignant tumors of the trachea are rare, and the best treatment has not yet been determined (16–19). Among the patients who visited our hospital, only 65.8%(52 patients) were diagnosed with new cases, and the remaining patients were referred to our hospital for various reasons. Most patients underwent various treatments, and only 16 patients (20.3%) were treated with only one treatment regimen. Forty-one patients (51.9%) were treated with ≥ 3 types of treatment, 15 (19.0%) were treated with ≥ 4 types of treatment, and 2 patients (2.5%) were treated with ≥ 5 types of treatment. Because most patients were treated with different types of treatment, grouping patients according to treatment type was impossible, thus precluding a survival analysis and comparisons of treatment plan side effects.

Survival analysis for the entire cohort revealed that the PFS and OS of patients who consumed alcohol regularly were significantly shorter than those of patients who did not drink alcohol or who occasionally consumed alcohol. However, the COX regression analysis did not demonstrate a statistically significant association. The specific reason for this observation may be attributed to the limited number of cases involving regular alcohol consumption, with only three such patients included in the study cohort. All 3 of these patients had SCC and a long history of smoking. The disease was located in the thoracic trachea. All of them were diagnosed with new cases and visited our hospital. One patient had stage E2N1M1 disease at the time of treatment and a history of esophageal cancer, chronic obstructive pulmonary disease, pulmonary tuberculosis, and pulmonary infection. This patient underwent ablation (argon-helium knife) + chemotherapy (albumin paclitaxel + lobaplatin) + immunotherapy (Sindili monoclonal antibody). During treatment, the patient developed a tracheoesophageal fistula, indicating an extreme cachexia state, and the family refused follow-up chemotherapy and immunotherapy. The other patient had stageE1N1M0 disease at the time of treatment and a history of esophageal cancer. This patient was also treated with ablation (helium knife) + chemotherapy (albumin, paclitaxel + nedaplatin) + immunotherapy (carrelizumab). A tracheoesophageal fistula also developed during the course of treatment. Another patient was diagnosed with stage E1N0M0 disease at the time of treatment. He had a history of esophageal stent placement, gastric fistula, craniocerebral surgery, and an electrolyte disorder. He underwent balloon dilatation of the trachea and tracheal stent placement to relieve symptoms. After the patient refused radiotherapy and chemotherapy, he was discharged, and no antitumor treatment was given. These 3 patients had a history of other diseases, and 2 patients with esophageal cancer developed a tracheoesophageal fistula after treatment, resulting in a poor prognosis. The other patient did not receive corresponding antitumor treatment, and the prognosis was poor. Only 3 patients reported regularly drinking alcohol, but this small number may have been because the clinician did not inquire in detail about the patients’ drinking histories at the time of medical history documentation and “nondrinker” is the default response, leading to deviation in the data. Therefore, whether regular alcohol consumption impacts survival outcomes requires further investigation with a larger sample size.

Although smoking did not affect the survival of ACC and SCC patients, it did affect the PFS and OS of patients with other pathological types. However, the COX regression analysis did not reveal a statistically significant association. Consequently, the impact of smoking on survival outcomes necessitates further investigation with a larger dataset.

In this study, the T stage at the time of diagnosis had no effect on survival. This is different from the results of the study by Bhattacharyya et al. (29, 37–39). They believe that the extent of the primary tumor or whether adjacent structures and organs are involved is related to the patient’s prognosis. Moreover, studies have revealed that thyroid infiltration is reflective of a poor prognosis in SCC patients (29, 40), and T stage is also an important prognostic factor for tracheal ACC (29, 41). However, our study did not confirm the effect of T stage on prognosis.

In addition, in this study, the N stage at the time of treatment affected the DMFS of the entire group of patients. The COX regression analysis further demonstrated that a higher N stage is significantly associated with an elevated risk of distant metastasis. However, because data are limited, the pathological type with the most impact on survival remains unclear. In patients with ACC and SCC, the N stage does not affect survival. There are different opinions on the effect of N stage on survival. The data presented by Piórek (29) revealed a statistically significant difference in OS between patients with and without lymph node involvement (37.7 vs.8.7 months), and there was a statistically significant difference in both 5-year OS (65% and 0%) and 3-year OS (73.1%and 16.7%).In the study by Bhattacharyya et al. (37), the survival of patients with regional lymph node metastasis was more than 50% shorter than that of patients without lymph node metastasis. In many studies, researchers have emphasized the risk of lymph node involvement and presented specific analyses of pathological types. Among ACC patients, 0–35.3% have lymph node metastasis, which has been found to be indicative of a poor prognosis in most studies (29, 42). In a large study including patients diagnosed with ACC from 1962 to 2007, the 5-year OS rate of N0 patients was 76%, and the 5-year OS rate of N1 patients was 54%(P = 0.017) (29, 43). In a study of SCC patients in the same center, the 5-year OS rates were 60%and 24%, respectively (P = 0.049) (40). A report from Massachusetts General Hospital also revealed that SCC with lymph node metastasis was associated with a lower survival rate, while there was no correlation between lymph node status in cases of ACC and survival (11, 28).

Univariate analysis indicated that the presence of distant metastasis at the time of initial consultation significantly affects both PFS and DMFS. However, COX regression analysis revealed that the presence of metastasis had no effect on survival. Distant metastasis is rare at the time of diagnosis of malignant tracheal tumors (29). Among the patients in this study, only 6.3%of ACC patients, 8%of SCC patients, and 4.5%of patients with other pathological types had distant metastasis at the time of treatment, all of which were similar to the proportions reported by Gaissert et al (38). However, few studies have evaluated the significance of the M stage in predicting the prognosis (29). Two of the largest studies (38, 42) and A. Piórek’s study (29) have revealed that the M stage has a negative impact on OS. Therefore, the influence of distant metastasis on survival outcomes requires further investigation with a more extensive dataset.

The 3-year overall survival rate was 69.9%, the 5-year overall survival rate was 62.3%, the 10-year overall survival rates were 34.2% and the median OS was 96 months. The 5-year overall survival rate was higher than that previously reported (14, 22, 44, 45). However, these findings are comparable to the results reported by Liu et al. (28) The 5-year overall survival rate was 77.2%. **Figure 4** shows that the overall survival of SCC patients was the shortest among all pathological types, and the difference was statistically significant. In regard to the patients’ general data, the patients differed in terms of age, sex, smoking status, and N stage, but these factors did not affect survival. In addition to our study, many reports have shown that SCC patients have shorter survival times than ACC patients do. Among the 578 patients with primary tracheal tumors (1973-2004) registered in the SEER database (14), the 5-year overall survival rate of the whole group was 27%, and the 5-year overall survival rate of ACC patients (74.3%) was higher than that of SCC patients (12.6%).Among the 92 patients with primary malignant tumors of the trachea in the study by Bhattacharyya (37), the survival rate of patients with SCC (mean survival time of 44.0 months, 5-year survival rate of 34%) was lower than that of patients with ACC (mean survival time of 115 months, 5-year survival rate of 78%).He also noted (37) that the prognosis and survival of patients with primary tracheal cancer depend largely on the histology of the primary tumor and that the survival rate of patients with tracheal ACC is significantly higher than that of patients with other tracheal malignancies. However, a report from The Second Affiliated Hospital of Air Force Medical University revealed that there was no significant difference in OS between ACC, SCC and other pathological types, whereas age and tumor size significantly affected OS (28).

In cases where radical surgery is not possible for tracheal malignant tumors, different endoscopic treatments or radiotherapies can be used (23). Most patients with malignant tracheal tumors in our center are from the respiratory hospital of our hospital. Our center is known for advanced technology, including stent placement under the guidance of flexible or rigid bronchoscopy. Therefore, most of the patients in this center have previously undergone endoscopic treatment. A total of 55.7% of the patients underwent bronchoscopic tumor resection, 51.9% of the patients underwent local ablation with an argon-helium knife, helium knife, argon knife, or Haibo knife, lesion freezing, microwave ablation, or electric knife cauterization to control the tumor, and 27.8% of the patients underwent close-range radiotherapy (iodine-125 particle implantation or radioactive stent implantation). Moreover, because most patients in our center are treated with various treatment methods, grouping patients according to the treatment plan was impossible, which is a limitation of this study. However, the overall survival of patients in this center was comparable to that reported in previous studies, which does not exclude the credit of endoscopic treatment. However, according to previous studies, endoscopic treatments, such as those involving lasers and stents, cryotherapy, and brachytherapy, are often considered palliative (2, 3, 46) as well as considered for unresectable tumors or patients with surgical contraindications (15). A study from Italy revealed that from April 1982 to December 1994, 2008 patients with malignant tumors obstructing the airway were treated with 2798 sessions of endoscopy alone and that 1838 patients underwent laser resection, 173 of whom underwent stent implantation, 29 received intracavitary brachytherapy, 133 underwent stent implantation, and 37 received brachytherapy alone. Finally, airway patency was achieved immediately after laser resection in 93%of patients, effectively improving their quality of life (47).

There are several limitations in this study. Owing to the diversity of treatments given, it was impossible to analyze the survival of the corresponding groups according to the treatment plan. In addition, some patients were previously treated in other hospitals and then transferred to our hospital for various reasons; thus, some data are missing. However, in addition to ACC and SCC, other pathological types were included in the present study.

In summary, our study confirmed that SCC is more common in patients who smoke frequently and are male, whereas ACC and other pathological types are more common in patients who are nonsmokers and female, and the overall survival prognosis of SCC patients is the worst of all pathological types. Most of the patients in this center receive a variety of treatments, thus it is impossible to ascertain whether the higher overall survival rate was due to this fact. Therefore, more studies with more patients are needed to ascertain the optimal treatment plan for malignant tracheal tumors.

## Data Availability

The raw data supporting the conclusions of this article will be made available by the authors, without undue reservation.
